# Integrated evidence reveals a new species in the ancient blue coral genus *Heliopora* (Octocorallia)

**DOI:** 10.1038/s41598-018-32969-z

**Published:** 2018-10-26

**Authors:** Zoe T. Richards, Nina Yasuda, Taisei Kikuchi, Taryn Foster, Chika Mitsuyuki, Michael Stat, Yoshihisa Suyama, Nerida G. Wilson

**Affiliations:** 1 Western Australian Museum, Welshpool, WA 6106 Australia; 2 Trace and Environmental DNA Laboratory, School of Molecular and Life Sciences, Curtin University, Bentley, WA 6845 Australia; 3 Organization for Promotion of Tenure Track, University of Miyazaki, Miyazaki, 889-2192 Japan; 4 Parasitology, Faculty of Medicine, University of Miyazaki, Miyazaki, 889-1692 Japan; 5 Australian Institute of Marine Science, Crawley, Perth, WA 6009 Australia; 6 Field Science Center, Graduate School of Agricultural Science, Tohoku University, 232-3 Yomogida, Naruko-onsen, Osaki, Miyagi 989-6711 Japan; 7 Department of Biological Sciences, Macquarie University, Sydney, 2109 Australia; 8 University of Western Australia, Crawley, Perth, Western Australia 6009 Australia

## Abstract

Maintaining the accretion potential and three dimensional structure of coral reefs is a priority but reef-building scleractinian corals are highly threatened and retreating. Hence future reefs are predicted to be dominated by non-constructional taxa. Since the Late Triassic however, other non-scleractinian anthozoans such as *Heliopora* have contributed to tropical and subtropical reef-building. *Heliopora* is an ancient and highly conserved reef building octocoral genus within the monospecific Family Helioporidae, represented by a single extant species – *H*. *coerulea*, Pallas, 1766. Here we show integrated morphological, genomic and reproductive evidence to substantiate the existence of a second species within the genus *Heliopora*. Importantly, some individuals of the new species herein described as *Heliopora hiberniana* sp. nov. feature a white skeleton indicating that the most diagnostic and conserved *Heliopora* character (the blue skeleton) can be displaced. The new species is currently known only from offshore areas in north Western Australia, which is a part of the world where coral bleaching events have severely impacted the scleractinian community over the last two decades. Field observations indicate individuals of both *H*. *coerulea* and *H*. *hiberniana* sp. nov. were intact after the 2016 Scott Reef thermal stress event, and we discuss the possibility that bleaching resistant non-scleractinian reef builders such as *Heliopora* could provide new ecological opportunities for the reconfiguration of future reefs by filling empty niches and functional roles left open by the regression of scleractinian corals.

## Introduction

Scleractinian corals are the principal engineers of modern-day shallow water tropical coral reefs. The scleractinians originated approximately 450 mya from a solitary and azooxanthellate ancestor^1^. But it was not until the middle Triassic (ca. 240 Ma) in tandem with dinoflagellate diversification events^2^ that the modern shallow-water Scleractinia underwent their first major radiations. Since that time, scleractinian corals have endured massive climate changes, but the modern combination of contemporary climate and anthropogenic impacts has surpassed coral tolerance limits, and at many locations, scleractinians are retreating^3^. The vulnerability of scleractinians threatens to jeopardize the accretion potential and productivity of coral reef ecosystems as a whole and as a result, coral reefs are predicted to transform in unprecedented ways. The most anticipated reconfiguration of future reefs involves transitions from hard coral to non-calcifying macroalgal communities or non-reef-building soft coral communities^4–6^.

Progressive shifts to altered reef states dominated by non-constructional taxa would jeopardize the biological functions and ecosystem services that coral reefs provide. Hence, maintaining the accretion potential and three dimensional structure of coral reefs is a priority^7^. In this regard, it is important to note that there is another, often overlooked genus of cnidarian that also contributes to reef-building – *Heliopora* de Blainville, 1830. *Heliopora coerulea* is one of the two extant members of the Anthozoan Order Helioporacea^8^ and the single extant member of the Family Helioporidae (Moseley, 1876). This species of octocoral, commonly called the ‘blue coral’ due to its distinctive blue fibrous aragonite skeleton, is unique among the Octocorallia because it is hermatypic. The species plays an important role in reef accretion in tropical locations^9^, and at some locations (e.g. Boliano Reef, Phillippines; Shiraho Reef, Japan; Bikini Atoll, Marshall Islands; Tarawa Atoll, Kiribati), blue coral is the dominant reef-building coral in shallow reef zones^10–13^.

*Heliopora coerulea* has a robust fossil record dating back approximately 120 million years to the lower Cretaceous^14^. It is often portrayed as a ‘living fossil’ because all of the morphological variation known from the fossil record is contained within the extant species^15^. The term living fossil has however, largely been abandoned because it does not reflect the genetic evolution within an organism^16,17^. No study has tested genetic structure across the entire range of *Heliopora*, but two cryptic lineages were recently identified along the Kuroshio Current, from the Philippines through to Taiwan and Japan^18–20^. Both of these lineages share the distinctive blue skeleton. At sites where the two lineages occur in sympatry, dissimilarities in habitat use, growth form, as well as reproductive timing were observed. These studies highlighted that additional diversity is likely to be present within the Family Helioporidae.

**Figure 1:**
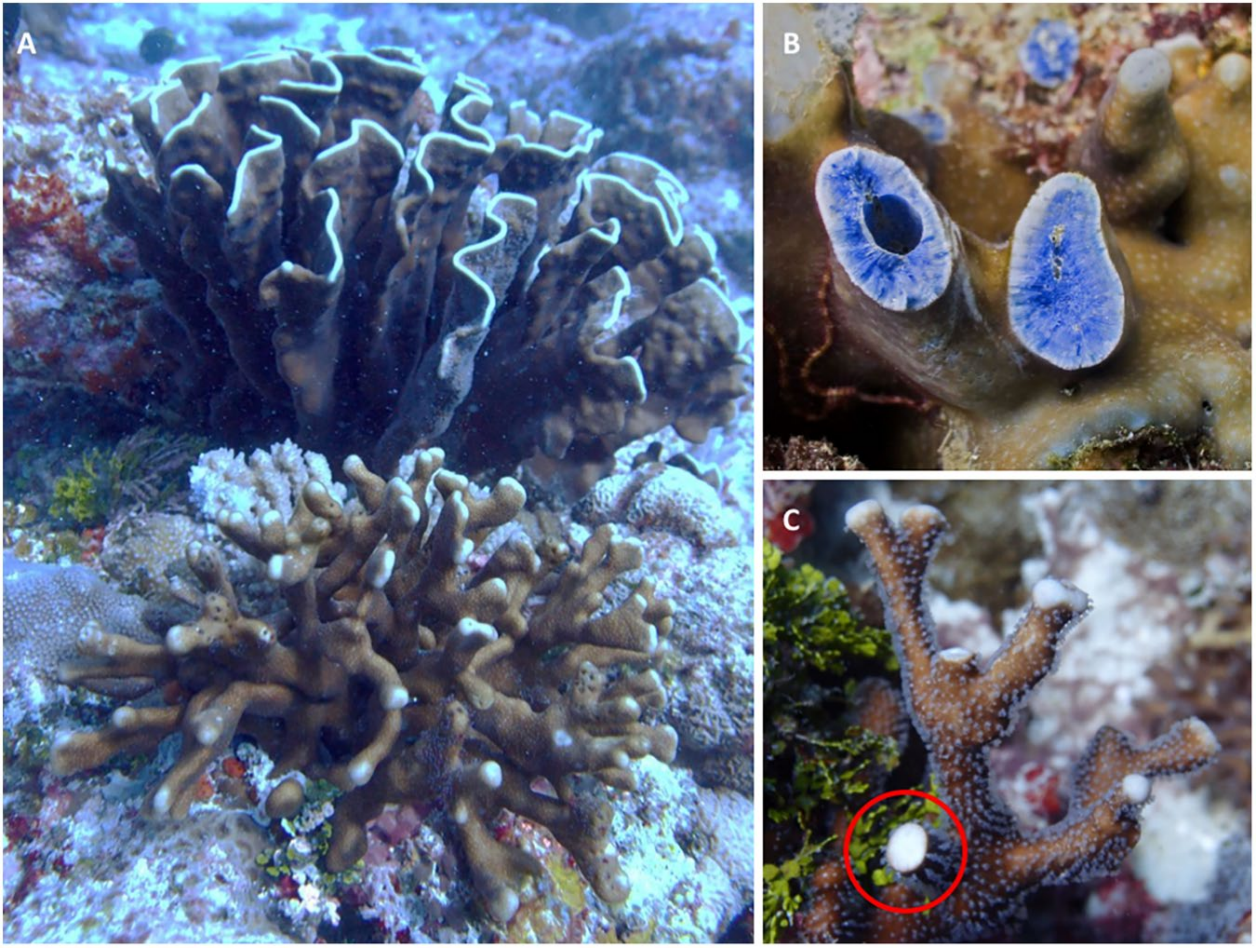
Two sympatric morphs of *Heliopora* occur in NW Australia. (**A**) The slender-branching *Heliopora hiberniana* sp. nov. (foreground) growing *in situ* with *Heliopora coerulea* (background) at the type locality, Hibernia Reef, NW Australia. (**B**) A broken branch reveals the characteristic blue skeleton of *H*. *coerulea*. (**C**) A broken branch (red circle) reveals the white skeleton of *H*. *hiberniana* sp. nov.

To examine the diversity of *Heliopora*, and explore the role that *Heliopora* may play on future reefs we performed an integrated systematic analysis of *Heliopora* in north Western Australia. The distribution and abundance of *Heliopora* was recorded on replicated belt transects at 165 sites. During these surveys an unusual, distinctive slender branching white morphotype was recorded (Fig. 1) (along with another slender branching ‘intermediate’ morphotype with blue streaks). We use a total evidence approach that included cladistic, phylogenetic (host/symbiont), population genetic, next generation high throughput genomic and reproductive analyses to explore the relatedness of the three morphotypes.

## Results

A total of 508 *H*. *coerulea* (blue) and 43 *Heliopora* sp. (white and intermediate) colonies were recorded across the survey area (Fig. S1). *Heliopora coerulea* occurred at inshore, midshelf and oceanic locations and is a dominant species in both subtidal and intertidal zones (Fig. S2). The white (and intermediate) *Heliopora* morphotypes occurred in shallow subtidal zones at 14 sites spanning the oceanic locations of Ashmore, Hibernia and Scott Reef (see Fig. S3). Most importantly, at Hibernia Reef the white and blue individuals were growing sympatrically (Fig. 1A).

### Morphology and reproductive biology

**Figure 2:**
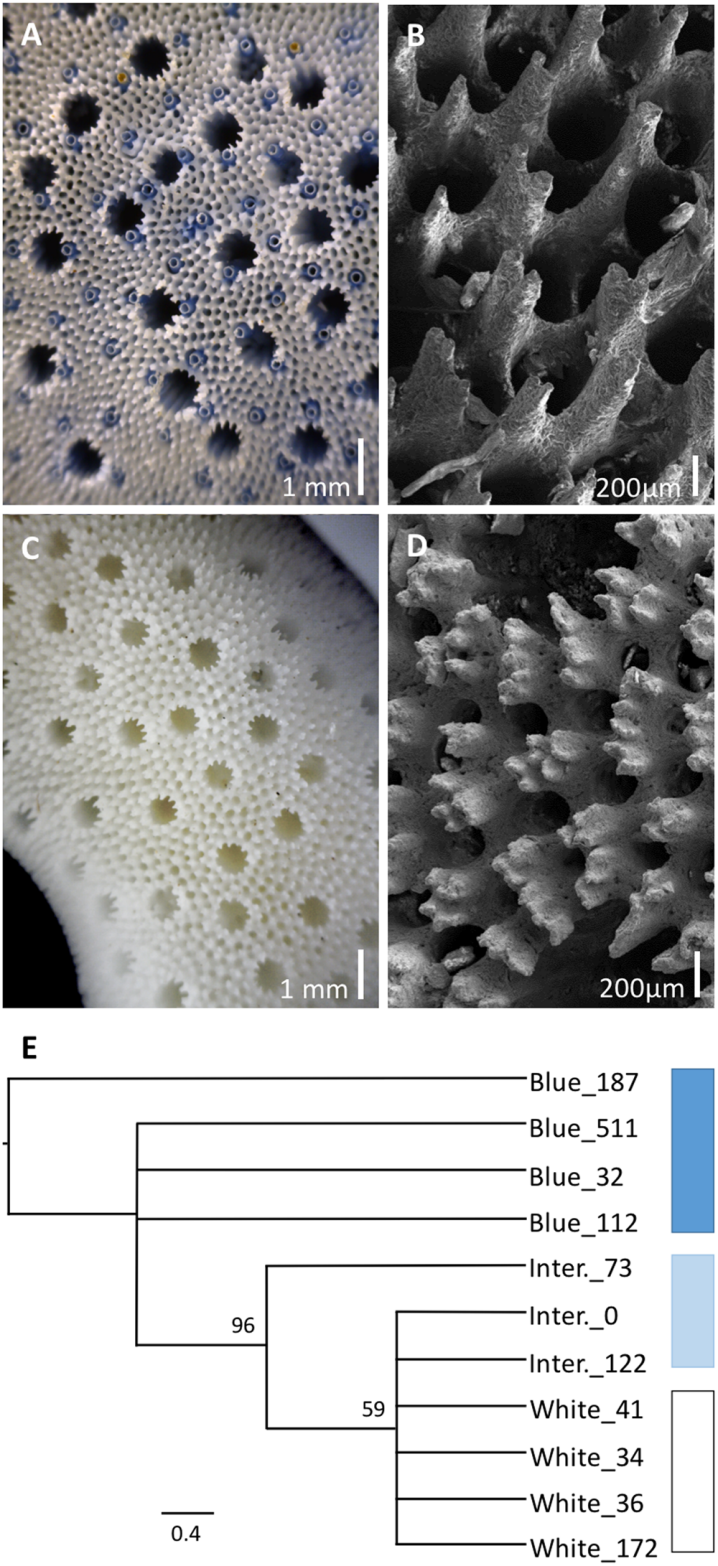
Comparative morphology of *Heliopora coerulea* and *Heliopora hiberniana* sp. nov. (**A**) Close up of *H*. *coerulea* showing the blue coloration of the skeleton. (**B**) Simple elaborations on echinulations in *H*. *coerulea*. (**C**) Close up of *H*. *hiberniana* sp. nov. showing the presence of autopores and absence of worm tubes. (**D**) Highly elaborated echinulations in *H*. *hiberniana* sp. nov. (**E**) Cladistic semi-strict consensus tree (of four equally-parsimonious trees) based on ten morphological characters confirms white and intermediate forms of *Heliopora hiberniana* sp. nov. are monophyletic and derived within blue *Heliopora coerulea*.

All specimens examined shared characteristic Heliopora morphological traits including the presence of autopores with pseudosepta, elaborated echinulations and siphonopores (Fig. 2, Table S1). A cladistic analysis based on ten morphological characteristics supported the differentiation of the white and intermediate morphs of *Heliopora*, which formed a highly supported clade (BS 96) (Fig. 2E). Key character states distinguishing the white and intermediate morphotypes from the blue *Heliopora* included slender branches; more elaborated echinulations, and smaller, more numerous autopores.

**Figure 3:**
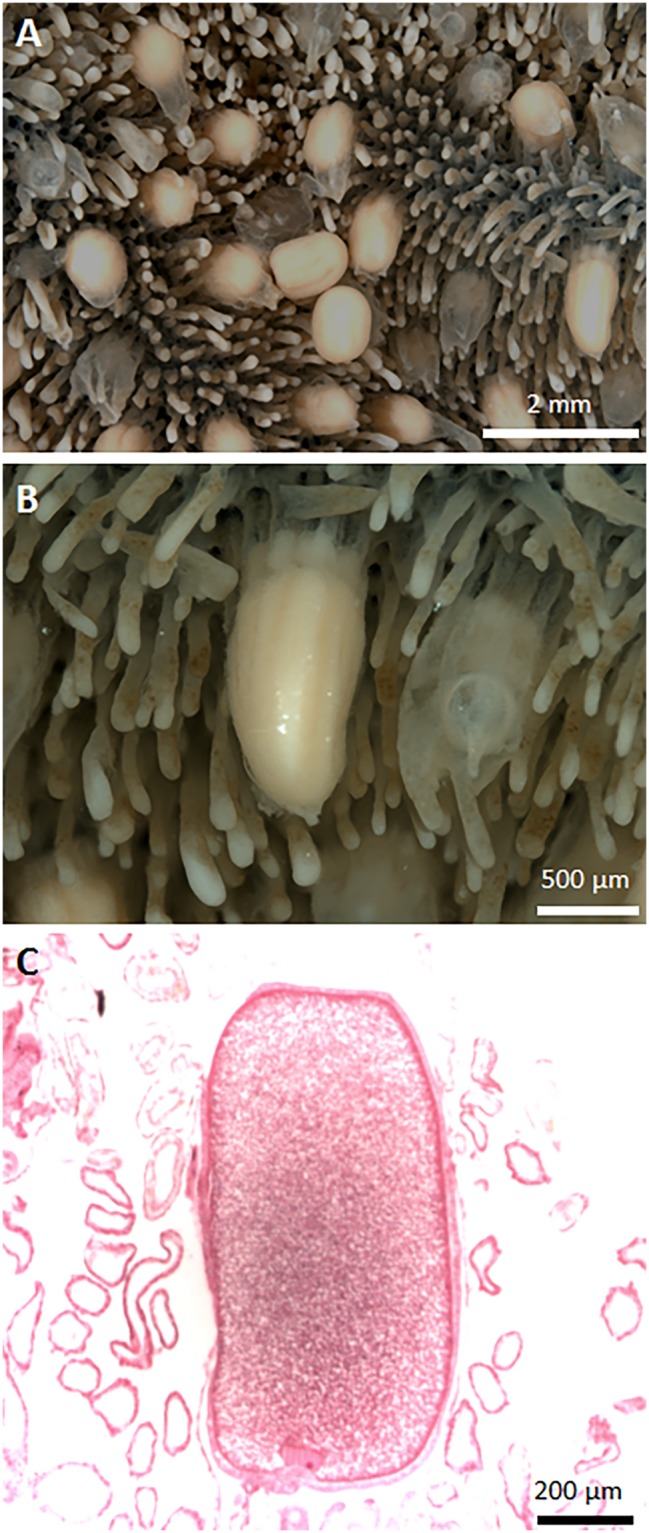
Reproductive biology of *Heliopora hiberniana* sp. nov. (**A**,**B**) Well-developed oocytes (>1 mm greater diameter) in the coelenteron which are larger than mean maximum egg sizes for scleractinians (recorded as 810 µm for *Lobophyllia hemprichii*, https://coraltraits.org); (**C**) Histological section of a well-developed oocyte showing the nucleus and lipid vacuoles. Considering *H*. *coerulea* is a surface brooder, and the planulae of this species have been shown to have a low lipid content^68^ the finding of such well-developed eggs still in the coelenteron raises the possibility that *H*. *hiberniana* sp. nov. may not exhibit surface brooding behavior.

Dissections of sympatric individuals of the white and blue morphotypes that were concurrently sampled from Hibernia Reef in Oct. 2013 revealed that all of the colonies of the slender branching white morph had either mature or well-developed eggs (>1 mm) in the coelenteron (Fig. 3A,B) while all *H. coerulea* colonies had either under-developed oocytes or were devoid of gametes. The existence of oocytes in the white morphotype (rather than planular larvae) was confirmed in histological section due to the presence of the nuclei, lipid vacuoles and the vitelline membrane (Fig. 3C).

### Phylogenetic and genotypic relationships

All *Heliopora* individuals in this study were identical based on sequences of the mitochondrial Cytochrome Oxidase I gene (COI) and the mitochondrial DNA mismatch repair protein Mutator S gene (mtMutS) (not shown). Nuclear Internal Transcribed Space 2 (ITS2) sequence data didn’t contradict the morphological cladogram, with white and intermediate individuals clustering into a derived clade (BS 59) within blue *Heliopora* (Fig. S4). Deep ITS2 amplicon sequencing revealed that all *Heliopora* individuals contained clade C *Symbiodinium* and that the OTU represented by ITS2 type C1 accounted for 95.2% of all sequences, (Fig. S5).

**Figure 4:**
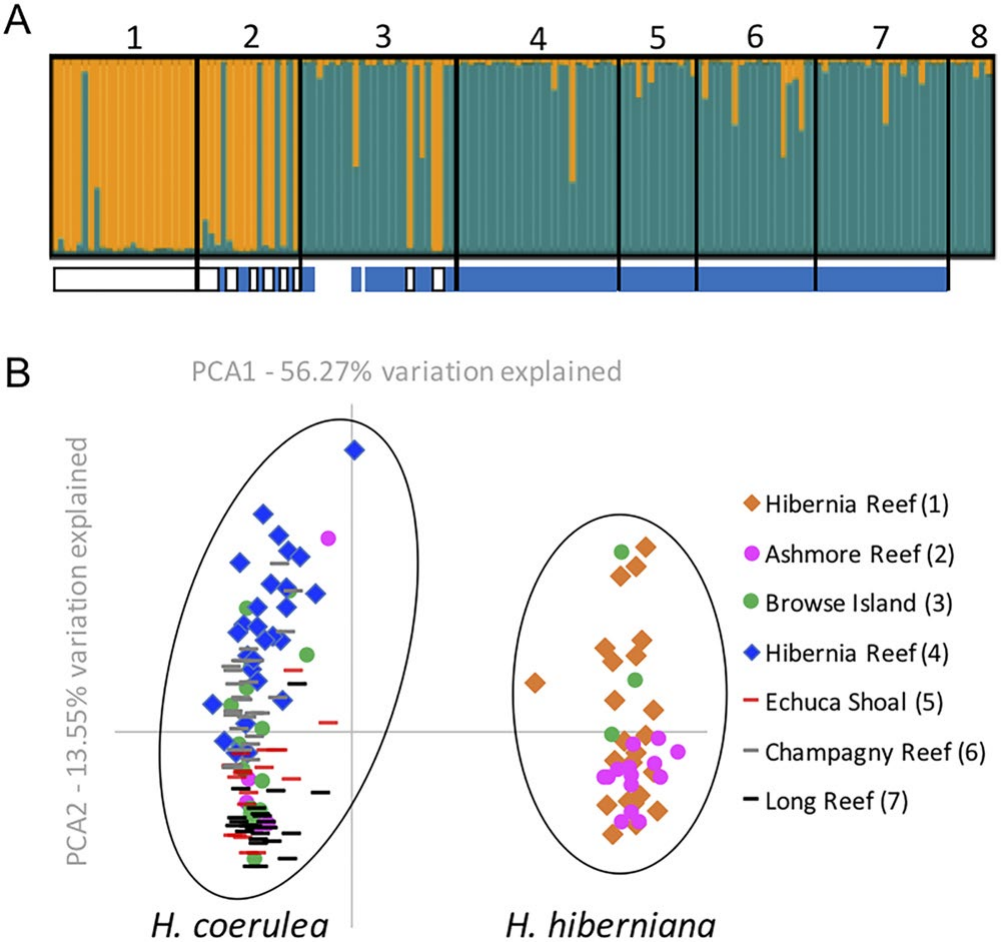
Microsatellite and MIG-seq data showing two clusters within *Heliopora*. (**A**) No a priori STRUCTURE plot for microsatellites confirms there are two lineages. Blue and white panels below STRUCTURE plot show where these individuals are assigned in the MIG-seq data. White panels indicate individuals clustering with *H*. *hiberniana* sp. nov., blue panels indicate individuals clustering with *H*. *coerulea*. *Heliopora hiberniana* sp. nov. (1) is differentiated from the sympatric *H*. *coerulea* population (4) at the type locality – Hibernia Reef, but sympatric with *H*. *coerulea* populations from Ashmore Reef (2) and Browse Island (3). Population 1 is white, populations 2–8 are blue. Population 8 = Christmas Island was not included in the MIG-seq analysis. (**B**) Next-generation MIG-seq analysis of individuals confirms and clarifies the group assignments in the microsatellite analysis.

Further examination of genotypic relationships within *Heliopora* using microsatellite markers in a population genetic framework confirmed that sympatric blue and white individuals from Hibernia Reef were strongly genetically differentiated (FST 0.379, Fig. 4A, Table 1). However, the genotypes of some blue individuals from Ashmore Reef and Browse Island grouped with the white individuals from Hibernia Reef, and a few other individuals appeared admixed or potentially mis-assigned. We then examined most individuals in a genome wide SNP analysis using multiplexed intersimple sequence repeats (ISSRs). An individual-based Principle Coordinate Analysis (PCoA) of the SNP data showed two clear clusters with no evidence of overlap, explaining approximately 70% of variation (Fig. 4B). The white individuals from Hibernia Reef occurred in one cluster and the blue individuals from Hibernia Reef occurred in the other, congruent with the patterns from the microsatellite data. Individuals that were not resolved by microsatellite data were clearly placed with the SNP data.

**Table 1:** Pairwise F_ST_ values (below) and P-values (above) calculated based on microsatellite data using Arlequin V 3.5 with possible clones excluded.

	ASH	BRI	CHM	ECH	HBL	LNG	XMAS	HIB
ASH		<0.001	<0.001	<0.001	<0.001	<0.001	<0.001	0.283
BRI	**0**.**19**		<0.001	<0.001	<0.001	<0.001	0.746	<0.001
CHM	**0**.**275**	**0**.**137**		0.042	0.005	<0.001	0.029	<0.001
ECH	**0**.**275**	**0**.**151**	0.057		<0.001	0.177	0.134	<0.001
HBL	**0**.**26**	**0**.**084**	**0**.**061**	**0**.**184**		<0.001	0.007	<0.001
LNG	**0**.**29**	**0**.**114**	**0**.**108**	0.03	**0**.**183**		0.154	<0.001
XMAS	**0**.**255**	−0.02	**0**.**103**	0.083	**0**.**129**	0.057		<0.001
HIB	0.11	**0**.**304**	**0**.**385**	**0**.**383**	**0**.**379**	**0**.**377**	**0.407**	

ASH = Ashmore Reef (*H*. *coerulea*, blue); BRI = Browse Island (*H*. *coerulea*, blue); CHM = Champagny Island (*H*. *coerulea*, blue); ECH = Echuca Shoals (*H*. *coerulea*, blue); HBL = Hibernia Reef (*H*. *coerulea*, blue); LNG = Long Reef (*H*. *coerulea*, blue); XMAS = Christmas Island (*H*. *coerulea*, blue); HIB = Hibernia Reef (*H*. *hiberniana* sp. nov., white).

From the combined morphological and molecular results, we infer that the slender branching morphotypes (which includes all white individuals as well as some blue) comprise a new species herein described as *Heliopora hiberniana* sp. nov.

### Systematics

Subclass OCTOCORALLIA Haeckel, 1866

Order HELIOPORACEA Bock, 1938

Family Helioporidae Moseley, 1876

Genus *Heliopora* de Blainville, 1830

Diagnosis as for Family. Massive skeleton of crystalline aragonite, polyps in cylindrical tubes, interconnected via solenia.

Type species *Heliopora coerulea* Pallas, 1766.

*Heliopora hiberniana* sp. nov.

LSID urn:lsid:zoobank.org:pub:3A8C6974-7F2F-4D6E-B932-DAB55A8F7B27

Etymology: Latin, feminine, in reference to the type locality, adjectival form of Hibernia.

Distribution: Hibernia Reef, Ashmore Reef, Scott Reef, Browse Is., NW Australia.

### Material examined

#### Holotype

Z66417. WA Museum. Hibernia Reef, Station144. 11°58.4424′S 123°19.3248′E. 12 m, coll. Z. Richards, 5 Oct 2013.

#### Paratypes

Z66400. WA Museum. SW Hibernia Reef, Station 142. 11°59.2890′S 123°20.1510′E. 10 m, coll. Z. Richards, 4 Oct 2013; Z66411, WA Museum. Hibernia Reef, St.143. 11°57.7002′S 123°22.7148′E. 12 m, coll. Z. Richards, 5 Oct 2013.

#### Other material examined from the type locality

Z66410, Z66412, Z66419, WA Museum. Hibernia Reef, Station 143. 11°57.7002′S 123°22.7148′E, 12 m, coll. Z. Richards, 5 Oct 2013.

Z66401, Z89293, Z89299, WA Museum. Hibernia Reef, Station 142. 11°59.2890′S 123°20.1510′E, 10 m, coll. Z. Richards, 4 Oct 2013.

#### Other material examined

Z66283, WA Museum. Ashmore Reef, Station 129. 12°11.0352′S 123°06.0378′E, 12 m, coll. Z. Richards, 27 Sept 2013.

Z66309, WA Museum. Ashmore Reef, Station 130. 12°11.3088′S 123°07.7322′E, 12 m, coll. Z. Richards, 29 Sept 2013.

Z66318, Z66319, WA Museum. Ashmore Reef, Station 132. 12°10.3782′S 123°03.6696′E, 12 m, coll. Z. Richards, 29 Sept 2013.

Z66329, WA Museum. Ashmore Reef, Station 134. 12°16.4868′S 122°58.8768′E, 12 m, coll. Z. Richards, 30 Sept 2013.

Z66379, WA Museum. Ashmore Reef, Station 141. 12°12.6126′S 123°08.6436′E, 1 m, coll. Z. Richards, 03 Oct 2013.

Z89297, WA Museum. Ashmore Reef, Station 143. 12°11.6688′S 123°3.0090′E, 12 m, coll. Z. Richards, 01 Oct 2013.

Z89301, WA Museum. Ashmore Reef, Station 130. 12°11.3088′S 123°07.7322′E, 12 m, coll. Z. Richards, 29 Sept 2013.

Z89303, WA Museum. Ashmore Reef, Station 127. 12°14.2368′S 123°9.6024′E, 12 m, coll. Z. Richards, 03 Oct 2013.

Z89306, WA Museum. Ashmore Reef, Station 126. 12°11.0352′S 123°6.0378′E, 12 m, coll. Z. Richards, 29 Sept 2013.

Z89314, Z89315, Z89317, Z89319, WA Museum. Scott Reef, Station SS1. 14°1.424′S 121°51.623′E, 3 m, coll. Z. Richards, 04 Oct 2015.

### Skeletal characteristics

#### Skeletal characteristics of the holotype

**Figure 5:**
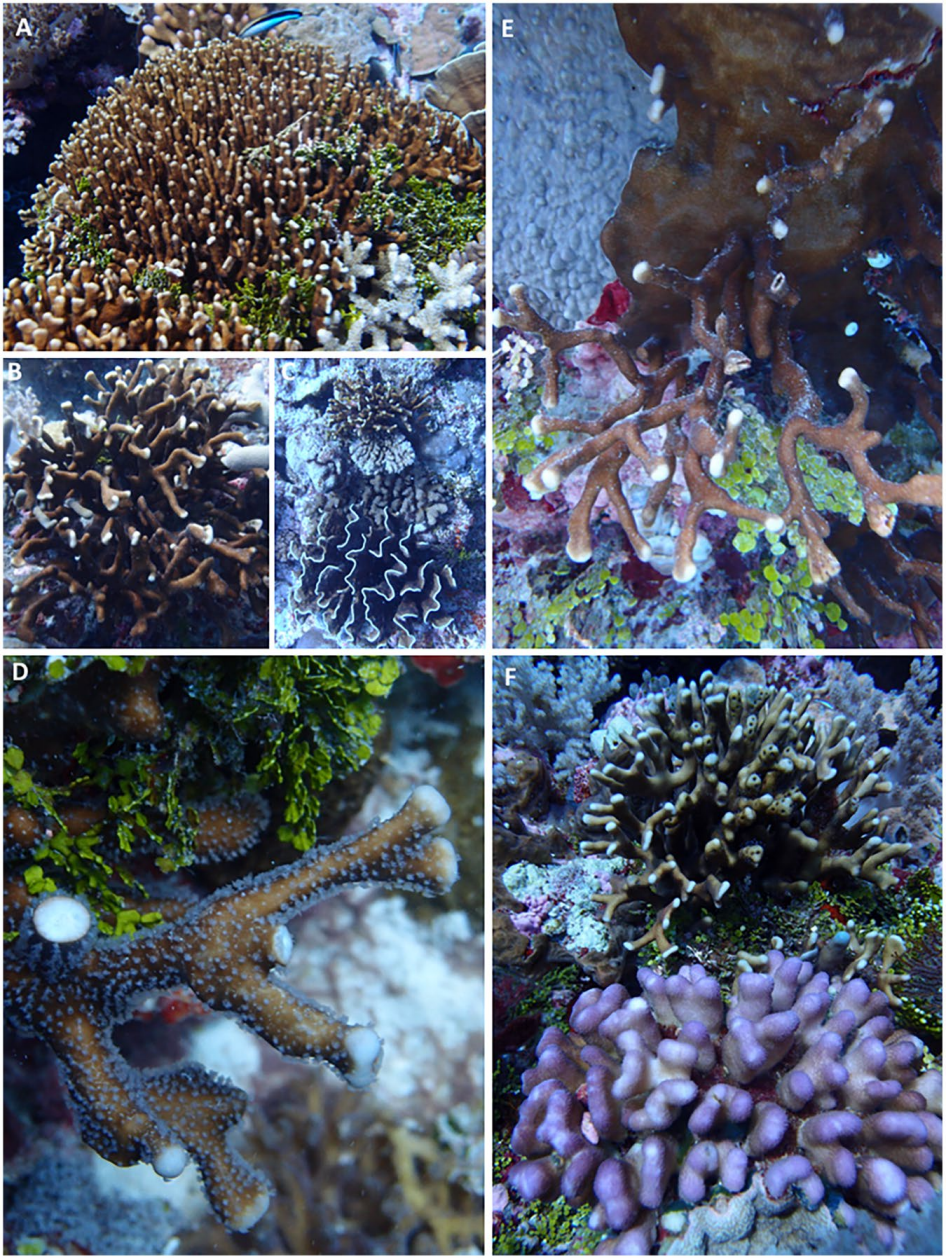
*Heliopora hiberniana* sp. nov. growing *in situ* at the type locality, Hibernia Reef. (**A**) Branching clump growing in close association with *Halimeda* sp., (**B**) Small branching clump. (**C**) *Heliopora hiberniana* sp. nov. (top) growing *in situ* with *H*. *coerulea* (bottom) at the type locality. (**D**) *Heliopora hiberniana* sp. nov. with a broken branch showing the white skeleton, (**E**) Side attached open branching colony with encrusting base; (**F**) *Heliopora hiberniana* sp. nov. (background) growing *in situ* with *Stylophora pistillata* in the foreground.

*Corallum – gross morphology*. Tightly branched clump, maximum colony diameter 40 cm. Main branches club shaped, irregularly dividing up to 30 cm long. Branches <15 mm wide and round in cross section at base with a tendency to flatten near tips (Figs 1 and 5).

**Figure 6:**
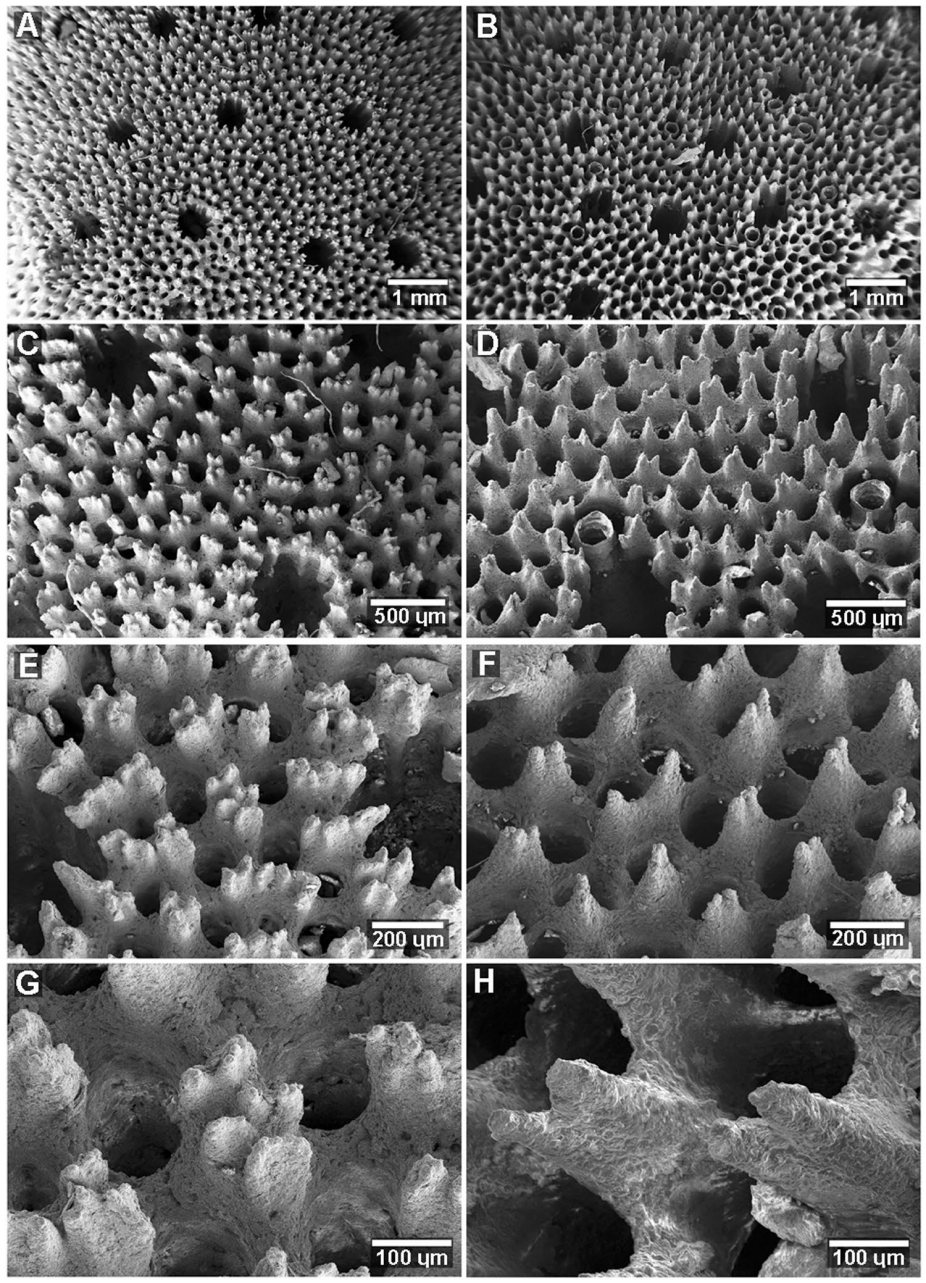
Scanning electron microscopy comparing the microstructure of *Heliopora hiberniana* sp. nov. (left panel) and *Heliopora coerulea* (right panel). Panels A and C, Holotype, Z66417; Panels B, D and F, Z89304; Panels E and G, Paratype Z66411; Panel H, Z89313.

*Macrostructure* and *microstructure*. The number of autopores varies greatly across the colony ranging from 9–17 per 5 mm^2^. Commensal worms absent (Fig. 2C, 6C). The autopore diameter ranges from 0.58–0.69 mm. Autopores are spaced from 0.65–1.53 cm apart. Pseudosepta 12–15 per autopore. Coenchymal echinulations are densely packed, 18–25 per 1 mm^2^ with a similar number of siphonopores (solenial tubes). The coenchymal echinulations are highly elaborated (3–6 elaborations per echinulation) (Figs 2D and 6E,G). See Table S1 for details of methods and replication.

#### Diagnosis

*Heliopora hiberniana* sp. nov. is distinguished from *H*. *coerulea* by a slender branching growth form, smaller and more numerous autopores, and highly elaborated echinulations. Some colonies (like the type material) are clearly distinguished by the presence of a white skeleton however this does not appear to be a fixed diagnostic trait as some *H*. *hiberniana* sp. nov. individuals retain the blue or intermediate colouration. *H*. *hiberniana* sp. nov. is distinguished from *H*. *compressa* Verrill, 1864 by its fine branching clump growth form and highly elaborated echinulations. *Heliopora compressa* is described to have a thick, massive or encrusting base that forms plates with thin edges or lobe-like branches. It also has 2–3 elaborations per echinulation rather than 3–6 as in *H*. *hiberniana* sp. nov. *Heliopora hiberniana* sp. nov. is distinguished by *H*. *fijiensis* Hoffmeister, 1945 by its fine branching clump form, smaller autopores (0.58–0.69 mm) and smaller number of pseudosepta (12–15). *Heliopora fijiensis* is known only from fossil material and it is described as having an encrusting growth form with 14–17 pseudosepta and an autopore diameter of 0.75–0.9 mm. The number of elaboration on echinulations were not recorded.

#### Variation based on paratypes

The paratypes show less variation in the number of autopores per 5 mm^2^ than the holotype however the range of variation falls within the variation expressed in the holotype (10–17). Worm tubes are absent. The autopore diameter measured in paratypes ranges from 0.41–0.65 mm indicating autopores can be smaller than those of the holotype. The spacing of autopores in the paratypes falls within the variation expressed in the holotype (0.82–1.24 cm). There are 10–15 pseudosepta per autopore in paratype material. There are a variable number of coenchymal echinulations (16–26 per 1 mm^2^) and siphonopores (solenial tubes, 21–28 per 1 mm^2^). As seen in the holotype, the coenchymal echinulations are highly elaborated with 3–6 elaborations per echinulation. See Table S1 for details of methods and replication.

#### Integrated comparisons among all examined material

Ten morphological characters were examined (Table S1) across eleven specimens (Table S2) and a matrix of the character codes was created for the cladistic analysis (Tables S3, S4). A cladistic analysis showed 96 bootstrap support for the intermediate and white morphs clustering together in a clade (Fig. 2). Significance testing on a subset of seven morphological characters verified that there was a significant difference in five character states between the three morphs (Table S5), however all seven characters were significantly different when the white and intermediate morph samples were pooled together (Table S6). Strict synapomorphies between the white and intermediate morphs include aspects of gross morphology, autopore diameter, the number of coenchymal echinulations and cavities, and the number of elaborations per echinulation (Table S5). Further examination of the blue morphotypes of *H*. *hiberniana* sp. nov. at Browse Island and Ashmore Reef will allow elucidation of the extent of morphological variation within the new species.

#### Distribution

*Heliopora hiberniana* sp. nov. is currently known only from four locations – Hibernia Reef, Ashmore Reef, Browse Island, and Scott Reef, north Western Australia (Fig. S3). It was observed growing between 1–12 m depth.

#### Reproduction

All of the five colonies of *H*. *hiberniana* sp. nov. that were examined for reproductive condition had mature oocytes in the coelenteron (Fig. 3). In comparison, colonies of *H*. *coerulea* had either less well-developed oocytes or were devoid of gametes. Considering *H*. *coerulea* is a surface brooder, the finding of such well-developed eggs still in the coelenteron suggests *H*. hiberniana sp. nov. may not be a surface brooder. Given the *H*. hiberniana sp. nov. specimens have narrow branches it is possible that surface brooding is not a viable life history strategy because larvae would be more prone to disturbance from currents, waves and/or other fauna than they are on *H*. *coerulea*, which tends to form large flattened blade-like fronds or thicker columnar colonies.

#### Remarks

There are only three available names of *Heliopora* listed in the World Register of Marine Species: *H*. *coerulea* (Pallas, 1766), *H*. *fijiensis* Hoffmeister, 1945 † and *H*. *compressa* Verrill, 1864. All are differentiated from *H*. *hiberniana* sp. nov. by morphology. *Heliopora fijiensis* remains known only from fossil material, and *H*. *compressa* is considered a *nomen dubium*^21^.

The new species was observed growing in close association with *Halimeda* sp. at the type locality (Fig. 5). Squat lobster *Alpheus obesomanus* Dana, 1852 (Arthropoda; Crustacea; Malacostracea; Decapoda; Alpheidae) were observed residing in colony tips (Fig. 5F).

## Discussion

Herein we report the finding of a new species, *Heliopora hiberniana* sp. nov. from recent surveys in north Western Australia. The divergence of *H*. *hiberniana* sp. nov. from *H*. *coerulea* is evident in the morphological cladogram and not conflicted by nuclear ITS2 sequence data, however similar to previous work^22^ we found all individuals of *H*. *hiberniana* sp. nov. and *H*. *coerulea* had identical mitochondrial sequences. In order to obtain a higher level of robustness, we added microsatellite and genome-wide ISSR data to test support for the existence of two species. Fundamentally, the test of the biological species concept was upheld here, as morphologically and genetically differentiated individuals representing the two putative species, were found sympatrically at Hibernia Reef.

The finding that some individuals of *H*. *hiberniana* sp. nov. feature a white skeleton indicates that the most diagnostic and conserved *Heliopora* character (the blue skeleton) can be labile. There is however, no indication in the fossil record that the blue skeletal colouration is variable, and this suggests that colour trait lability may have evolved under contemporary environmental conditions. Understanding how this character change has occurred requires exploring why blue coral is blue. The distinctive blue colour of *H. coerulea*’s crystalline aragonite skeleton is thought to be caused by the production of biliverdin IXa^23^. Biliverdin IXa is formed by the oxidative stress-inducible proteinheme oxygenase (HO) during heme decomposition^24^. A metatranscriptome analysis has confirmed the functional genes for heme synthesis are present in both *Heliopora coerulea* and its photosynthesizing symbiont^25^. However, the final part of the heme synthesis pathway, which converts biliverdin IXa to bilirubin via biliverdin reductase, was not identified. The absence of biliverdin reductase could be the reason why blue pigment accumulates in *Heliopora*. It is possible that *H*. *hiberniana* sp. nov. may have regained the ability to reduce biliverdin IXa, resulting in some individuals with partly or entirely white skeletons.

Considering the unique role that the symbiont plays in the c5 pathway leading to heme synthesis^25^, we also examined if there were any differences in symbiont species/lineages between *H. coerulea* and *H*. *hiberniana* sp. nov. However, all hosts contained clade C1 *Symbiodinium* indicating there is no detectable co-evolution between the host and symbiont that could explain the divergence. It is also possible that other coral biomineralization-related genes such as carbonic anhydrases, bicarbonate transporters, calcium-binding proteins or skeletal organic matric proteins may also be playing a role^26^ and further genomic studies are needed to understand the mechanism of colour character release.

Although the presence of white individuals helped distinguish *Heliopora* *hiberniana* sp. nov. species in the first instance, we also found that some blue individuals (from Ashmore Reef and Browse Island) grouped genetically with the white individuals of *H*. hiberniana sp. nov. from Hibernia Reef. The microsatellite data generally recovered two groupings, but indicated some level of mis-assignment of individuals. However, the final number of loci included was low (n = 5) and the number of alleles these recovered was also generally very low (Table S7), fundamentally reducing the power of the microsatellite data to resolve many individuals. Thus, we rely more strongly on the signal from the SNP data derived from MIG-sequencing. Here, the number of loci was much higher (n = 166), and the data resolved all individuals into two strongly differentiated clusters, with no evidence of admixture among species. This analysis confirmed that blue skeletal colouration can still be present in some individuals of *H*. *hiberniana* sp. nov.

Thus our dataset indicates that genetic differentiation precedes colour differentiation, and we infer that reproductive isolation most likely explains the observed genetic differentiation of *H. coerulea* and *H*. *hiberniana* sp. nov. *Heliopora* *coerulea* is a gonochoric surface brooder with an annual gametogenic cycle^27^. When sympatric individuals of the two species were sampled at Hibernia Reef in October 2013, all of the colonies of *H*. *hiberniana* sp. nov. had either mature or well-developed eggs in the coelenteron (Fig. 3) while *H. coerulea* colonies had either under-developed oocytes or were devoid of gametes. Biannual spawning is commonly reported in numerous other coral species in the north-western Australia^28^ and the implications of this for genetic subdivision and speciation are beginning to be explored^29,30^. The differential reproductive development in *H*. *hiberniana* sp. nov. and *H. coerulea* observed here suggests the two species may have a pre-zygotic reproductive barrier such as disparate spawning times. Hence, reproductive isolation is likely a driving or reinforcing mechanism for speciation in this highly conserved lineage.

The discovery of a new species in this ancient and morphologically conserved lineage is highly unusual and raises the question of when this species diverged, which may shed light on the evolutionary catalyst. To answer this fully requires dating the divergence event, which we have not attempted in the present study. Octocorals utilize a mitochondrial DNA repair mechanism, resulting in very low rates of mutation^31,32^; this makes the recovery of a robust phylogenetic framework to constrain divergence events extremely difficult. More sophisticated data sources will be required to address this line of questioning. Nevertheless, in both the morphological and ITS2 phylogenetic reconstructions, *H*. *hiberniana* sp. nov. was derived within *H. coerulea* indicating it is more recently evolved. It will be important in the future to examine the relationships between *H. coerulea* and *H*. hiberniana sp. nov. and lineages HC-A and HC-B reported in^18,19^. By expanding both the breadth of populations sampled and the extent of genome sampled, we will improve our ability to understand phylogeny and character evolution in this important genus of reef-building corals.

**Figure 7:**
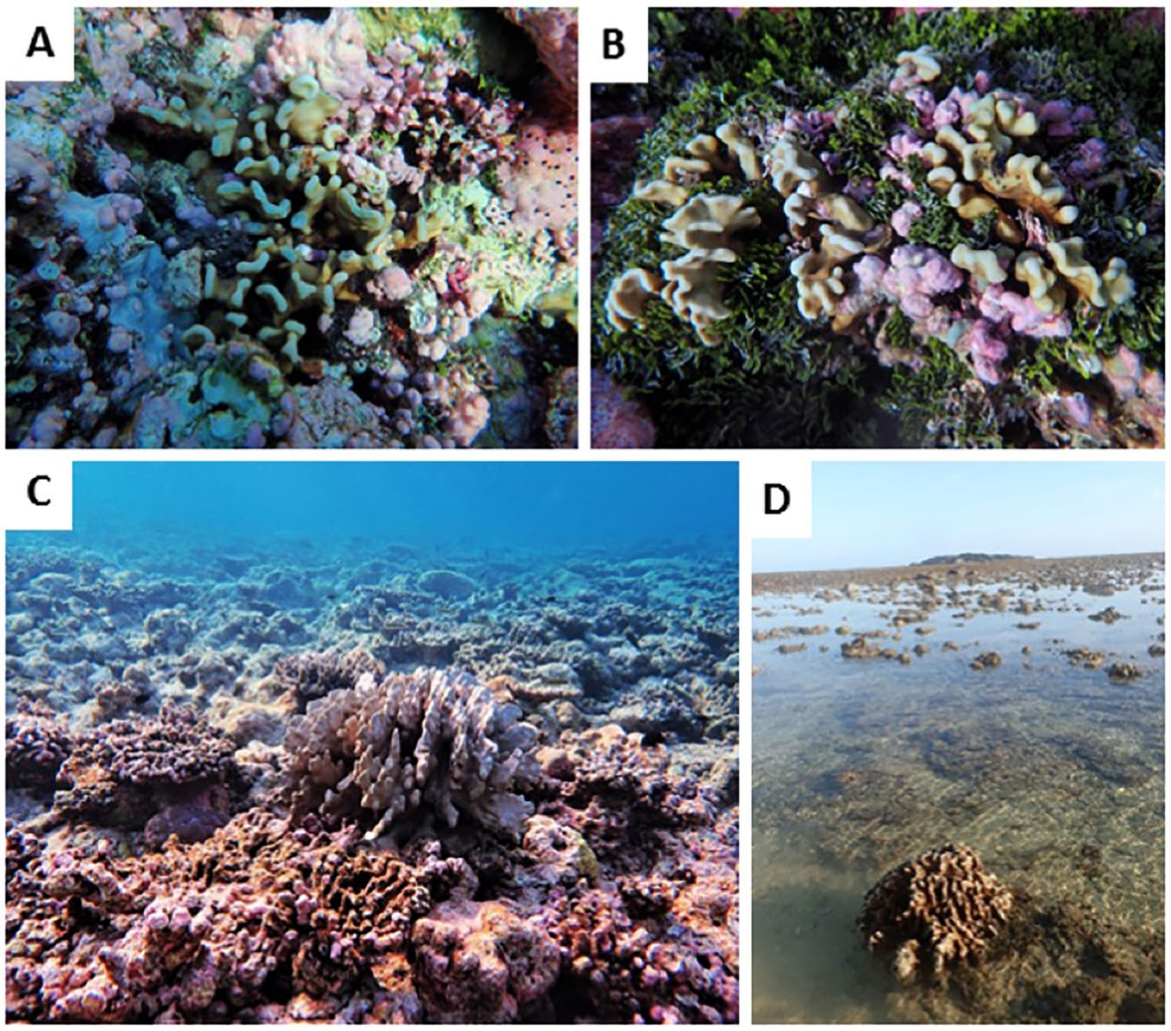
*Heliopora* is a resilient coral genus that has proven to be resistant to bleaching mortality^11,37–39^. For example, in 2016, coral communities at Scott Reef were devastated by coral bleaching^33^. When the status of the community was examined in a post-bleaching biodiversity survey in 2017, remnant colonies of both *H*. *hiberniana* sp. nov. (**A**, **B**) and *H*. *coerulea* (**C**) were intact. (**D**) *Heliopora coerulea* grows in a wide range of habitats including the hostile upper reef flat in the Kimberley, NW Australia.

It is also important to note that *H*. *hiberniana* sp. nov. has to date, only been located at offshore locations in north Western Australia, which is a region that was seriously impacted by the 1998^33^ and 2003^34^ bleaching events. Despite their persistence through ~ 450 mya of environmental variation^1^; contemporary scleractinian corals are highly threatened by climate change^35,36^. In 1998, between 80–90 percent of scleractinian coral at Scott Reef died resulting in a drastic reduction of the local brood stock and marked changes in community composition^33^. The fate of *Heliopora* was not recorded then, however in 2016 a second thermal stress event impacted coral communities at Scott Reef^35^ and post-bleaching surveys conducted in 2017 indicate both *H. coerulea* and *H*. *hiberniana* sp. nov. persisted through this disturbance event (Richards unpublished, Fig. 7). Numerous other studies suggest *H. coerulea* is highly resistant to bleaching mortality^11,37–39^ and transcriptomic studies suggest *Heliopora* has an abundance of transcripts with heat shock protein and antioxidant domains which may relate to bleaching resistance^26^. If *Heliopora* has a higher probability of persisting through future disturbance regimes it may become an increasingly important component of tropical Indo-Pacific coral reefs. Thus, our findings may herald the start of a unique period of ecological opportunity and change where non-scleractinian reef builders such as *Heliopora* rise up to fill niches left open by retreating scleractinians^40^ and play an important role in the reconfiguration of future reefs.

The retreat of scleractinians threatens to cripple coral reef ecosystem functioning and productivity and endanger the millions of people that rely on coral reefs for protection, nutrition and livelihood. Although the expansion of *Heliopora*, and other reef building Anthozoans such as *Millepora* may assist in the maintenance of critical ecosystem functions and provide a means for reef building, it is not clear if *Heliopora* calcification rates are high enough to maintain the positive carbonate budgets that are required to secure future reef growth^41^. In addition, a transition to *Heliopora* reefs may further impede scleractinian recovery because competitive exclusion via allopathy may inhibit the settlement of scleractinian coral juveniles^39^. This would also make it unfeasible to use *Heliopora* in the manipulative coral reef restoration approaches that are currently being explored^42^. Furthermore, the diversity of scleractinians cannot be matched by any other constructional anthozoan in the short-medium term, and the loss of scleractinian reef builders could have cascading impacts on associated marine biodiversity and unknown impacts on overall reef productivity.

## Methods

### Field Surveys

The diversity and abundance of *Heliopora* was examined on three replicate 15 m × 1 m belt transects at 165 sites covering a total of 8.265 km^−1^ in the Kimberley north Western Australia and Christmas Island (Fig. S1). Tropical reefs in the region range form diverse, tidally-driven systems in the inshore region^43^ to submerged platforms, midshelf shoals and oceanic shelf edge reefs and atolls^44^.

### Morphological Data Analyses

Measurements of skeletal morphological characteristics were calculated from Scanning Electron Microscopic (SEM) images taken at three random, non-overlapping areas on the surface of eleven coral samples. Three 2–3 cm fragments were cut from each individual and mounted cut-side-down onto glass slides with carbon tape and coated with 10 nm of platinum and 10 nm of carbon. Images were taken using a Zeiss 55 field emission SEM set to 5 kV.

Some of the white specimens had faint streaks of blue in the skeleton hence initially, corals were separated into three groups; ‘Blue’, ‘White’ or ‘Intermediate’. Replicate measurements of nine morphological characters (Table S1) were taken in four individuals of *Heliopora coerulea* (blue); four individuals of *Heliopora* sp. (white) and three individuals of *Heliopora* sp. (intermediate) (Table S2). In order to avoid pseudoreplication, the three measurements from individual coral colonies were averaged, and the means from each individual coral were used as replicates. All data met assumptions of normality using the Shapiro-Wilk test and assumptions of homogeneity of variance using Levene’s test for equality of variance. To examine if the morphological characters of the three groups were significantly different one-way ANOVA’s were conducted with ‘Blue’, ‘White’ and ‘Intermediate’ set as fixed factors using Tukey’s Honestly Significant Difference (HSD) post-hoc test. Based on the results of this test, individuals from the ‘White’ and ‘Intermediate’ groups were pooled together to form the pooled ‘White’ *Heliopora* group. Data were re-checked for normality and for equality of variance. The measure ‘autopore diameter’ did not meet the equality of variance assumption. For all the data that did meet the assumption of equal variance, independent samples T-tests were conducted to test for differences in the means between the ‘Blue’ and pooled ‘White’ groups (Table S6). For the measure ‘autopore diameter’, Welch’s T-test was used, as it does not assume equal variance. All tests were conducted in SPSS version 21^45^.

A morphological character matrix (Tables S3, S4) was used for cladistic analyses conducted in PAUP* 4.0a (build157)^46^. Sample 187 represented an all zero state for all characters and was used to root trees in the absence of an additional appropriate outgroup. Characters 1 and 6 were ordered, the rest were unordered, all equally weighted. Gaps were treated as missing, and a branch-and-bound search was utilised. Branch support was assessed with 1000 bootstrap pseudoreplicates with 100 random additions.

### Reproductive biology

Sampling was undertaken at site 145 at SW Hibernia Reef (S11.97605; E123.39967) on the 5th October 2013. Four-centimeter long branches were collected from six replicate colonies of blue and white *Heliopora* and fixed in 10 percent formalin/seawater, decalcified in a solution of 5 percent HCL and stored in 70 percent ethanol (Table S2). Gamete development was determined by the dissection of the decalcified polyps using a stereo-microscope. Oocytes were counted per 1 cm^2^ and measured along their longest axis and the axis perpendicular to that, using a micrometer. Photographs of dissected colonies were taken on a Leica MZ16A microscope and camera. Histological preparations were made of two representative samples from each colour morph. These were embedded in paraffin wax, sectioned at 6 um and stained with Haematoxylin and Eosin.

### Genetic Analyses

#### Direct sequencing of host

Sanger sequencing was conducted on 17 individuals (Table S2). Small fragments (1–2 cm) were preserved in 100 percent ethanol. Genomic DNA was extracted using a DNeasy Blood and Tissue Kit (Qiagen). Host variation was examined at two mitochondrial genes (COI and mtMutS) and one nuclear gene (ITS2) (Primers listed in electronic supplementary material, Table S7). Host COI and mtMutS sequencing followed the methods^22,47,48^ (PCR conditions are listed in Table S7). Host ITS2 sequencing was undertaken as in^22,49^ (see Table S7).

COI and mtMutS sequences were completely conserved and no further analyses was undertaken. The host ITS2 sequences were aligned with Sequencher. A maximum-likelihood analysis using RAxML v8^50^ was implemented with the GTR + G model, and branch support was assessed using 10 replicate runs of 1000 bootstrap replicates. A maximum parsimony analysis was also run on the same data set using PAUP* 4.0a (build 157)^46^. Characters were all unordered, equally weighted, optimised with ACCTRAN, using furtherest sequence addition. A branch-and-bound search recovered 3 most parsimonious trees, with the semi-strict consensus of these shown in Fig. 1C, using mid-point rooting. Branch support was assessed with 1000 heuristic bootstrap pseudoreplicates^51^ with 100 random additions and TBR branch-swapping.

#### Intragenomic sequencing of *Symbiodinium*

The *Symbiodinium* communities associated with blue (N = 6), white (N = 5) and intermediate (N = 5) *Heliopora* was investigated using deep sequencing of the nuclear ITS2 region. A single round of polymerase chain reaction (PCR) using fusion tag primers consisting of Illumina adaptor and sequencing primers, indexes unique to this study, and the template specific primers ITSD and ITS2rev2 (Table S7) was used to produce *Symbiodinium* ITS2 amplicons. PCR reagents included 1× AmpliTaq Gold® Buffer (Life Technologies), 2 mM MgCl_2_, 0.25 μM dNTPs, 10 μg BSA, 5 pmol of each primer, 0.12× SYBR® Green (Life Technologies), 1 Unit AmpliTaq Gold DNA polymerase (Life Technologies), 2 μl of DNA, and Ultrapure™ Distilled Water (Life Technologies) made up to 25 μl. PCR was executed on an Applied Biosystems StepOnePlus Real-Time PCR. The sequencing library was prepared by pooling PCR products (in duplicate) from each sample into equimolar ratios based on qPCR and quantification using a Labchip® GX Touch HT (Perkin Elmer), and sequenced using a 500 cycle MiSeq® v2 Reagent Kit and standard flow cell (2 × 250 paired end) on an Illumina MiSeq platform located in the TrEnD Laboratory at Curtin University.

Sequences were assembled using the Illumina MiSeq software under default settings, and assigned back to samples using a 100% identity match to index barcodes and the ITSD and ITS2rev2 sequences in Geneious® 8.1.4^52^. Mothur 1.36.1^53^ was used to remove singletons, sequences that had an average Q score ≤25, reads that contained ambiguous bases, and chimeras identified using Perseus^54^. The number of sequences per sample was sub-sampled to 40,000 reads, and sorted into OTUs at 97% similarity for each clade separately. A 97% cut-off was used because it has been shown to be appropriate for determining the diversity of *Symbiodinium* in ITS2^55^.

*Symbiodinium* OTU frequencies in each *Heliopora* colony were standardised, square root transformed, and used to produce a Bray-Curtis similarity matrix in PRIMER v.6^56^. A one-way Analysis of Similarity (ANOSIM) with colour morph as a factor was used to determine whether there was a significant difference in the community of *Symbiodinium* associated with the different *Heliopora* phenotypes, and visualized using a heatmap constructed using the gplots package in R^57^.

As *Heliopora* ITS2 host sequences were also recovered from the dataset, they were used to confirm haplotypes recovered by Sanger sequencing. A total of 343 host *Heliopora* ITS2 sequences were recovered from the Illumina dataset, viewed in Sequencher, and manually edited to align with the dominant allele in each individual. The dominant amplicon from each individual generated using NGS was identical to the amplicon recovered using Sanger sequencing and a total of seven polymorphic sites were observed in the 286 bp alignment.

### Population Genetics (Microsatellites)

Population samples (n = 12–30 individuals per population, Table S8) were collected from seven locations in the Kimberley and also from Christmas Island, which is an isolated oceanic island offshore from north Western Australia. Genomic DNA was extracted using a modified method by^58^. In short, 100 µL, of 50 mM NaOH was added in a tube with a coral fragment (less than 8 mm3), the sample was heated to 95 °C for 10 min. The tubes were then cooled to 4 °C, and 1/10th the volume of 1 M Tris-HCl (pH 8.0) was added to neutralize. The DNA was then cleaned and purified by ethanol precipitation.

Nine primers were initially examined for microsatellite analysis^18,59^ using the protocol of^18^. Five markers (miho04, saki06, saki08, emi20 and mayu41) amplified clear bands and were used for subsequent analysis. Identical multilocus genotypes were removed and basic statistics were calculated in Arlequin V 3.5^60^. A model-based clustering approach, implemented in STRUCTURE 2.3.4^61^ was used to examine how genetic variation was partitioned. The initial analysis which included all eight populations across multiple values for K (1–8) was undertaken with 20 independent runs for each value of K. The analysis was run using uniform priors [no LOC priors] and repeated with prior information based on skeletal colour. All runs included a 1,000,000 iteration burn in followed by 1,000,000 clustering iterations (MCMC). The analysis was also re-run for the sympatric Hibernia blue (HBL) and Hibernia white (HIB) populations with no prior information and with prior colour data.

### Population genetics (high-throughput genomic analysis)

We performed a multiplexed ISSR genotyping by sequence (MIG-seq) analysis, which is an effective PCR-based method for detecting genome-wide single-nucleotide polymorphisms (SNPs) to confirm genome-wide genetic differentiation^62^. The MIG-seq method amplifies anonymous genome-wide inter-simple sequence repeats (ISSRs) region^63,64^ using multiplex PCR without prior genetic information. The number of available informative loci from MIG-seq analysis is generally less than that using other techniques such as restriction site-associated DNA markers (RAD-seq)^65^. However, MIG-seq has several advantages: putatively neutral loci can be obtained because MIG loci are adjacent to microsatellite regions, the method can be performed on low-quality or small amounts of DNA, and is relatively easy to perform and cheap.

In total, 159 *Heliopora* samples from seven populations representing blue, white and intermediate individuals were selected to confirm the result of the microsatellite analysis (see electronic supplementary material, Table S6). The MIG-seq library was prepared for paired-end sequencing using the 8 pairs of multiplex primers (MIG-seq primer set-1) for 1st PCR. The DNA libraries from each sample with a different index were pooled and then clonally amplified on a flow cell following the protocol by^62^. Sequencing was performed using MiSeq (sequencing control software v2.0.12, Illumina) with MiSeq Regent v3 150 cycle kit (Illumina). Image analysis and base calling were performed using real-time analysis software v1.17.21 (Ilumina).

The FASTX toolkit was used to remove low-quality reads and primer sequence reads from the raw data using the following settings: a) the minimum percentage of individuals required to process a locus across all data (r) was set at 70 percent; b) a minimum coverage of 10 reads to create a stack (m) and, c) restricting data analysis to only the first SNP per locus to prevent possible linkage. Following sequence quality filtering, the program Stacks (v1.46) identified a total of 166 SNPs. In order to discriminate *Heliopora* genome sequences from symbiont or other genomic DNA, MiSeq [v3–600] was used to sequence 16 G bp of *Heliopora* genomic DNA extracted from symbiont-free larvae that were isolated in a previous study^29^. This genomic resource was used to confirm that the SNP loci (>15 read coverage) identified in this study were from *Heliopora* using the software SMALT (http://www.sanger.ac.uk/science/tools/smalt-0) with parameter settings −r 1 −x −y 0.8″ −k 20 −s 4.

Individual-based Principle Coordinate Analysis (PCoA) was performed using GeneAlex ver. 6.5 to examine genetic relationship between individuals. By using an admixture model and allele frequency correlated model, STRUCTURE analysis was carried out for 10 times each for values of K = 1–5, with 100,000 MCMC iterations following a burn-in period of 100,000. The most likely number of cluster delta K was determined using Structure Harvester^66^. Data files were converted to each software using PGDspider ver 2.0.8.3^67^.

## Electronic supplementary material

Supplementary Figures and Tables

## Data Availability

SNP data generated in this project are available from the Dryad Digital repository DOI:10.5061/dryad.50s5g5q. Genbank Accession numbers for ITS2 and mitochondrial sequences are listed in Table S2. Material examined in this study is housed at the Western Australian Museum and accession numbers are listed in Table S2.
