# Supplementary material for: Integrated evidence reveals a new species in the ancient blue coral genus Heliopora (Octocorallia)
Source: Sci Rep. 2018 Oct 26;8:15875. doi: 10.1038/s41598-018-32969-z (PMC6203795; doi:10.1038/s41598-018-32969-z)
Supplement: Supplementary file 1 — Supplementary Figures and Tables [file 41598_2018_32969_MOESM1_ESM.docx]

Supplementary Materials

Full Title: Integrated evidence reveals a new species in the ancient blue coral genus *Heliopora* (Octocorallia)

**Authors:** Zoe Richards^1,2^*, Nina Yasuda^3^, Taisei Kikuchi^4^, Taryn Foster^5^, Chika Mitsuyuki^6^, Michael Stat^2^, Yoshihisa Suyama^6^, Nerida Wilson^1,7^


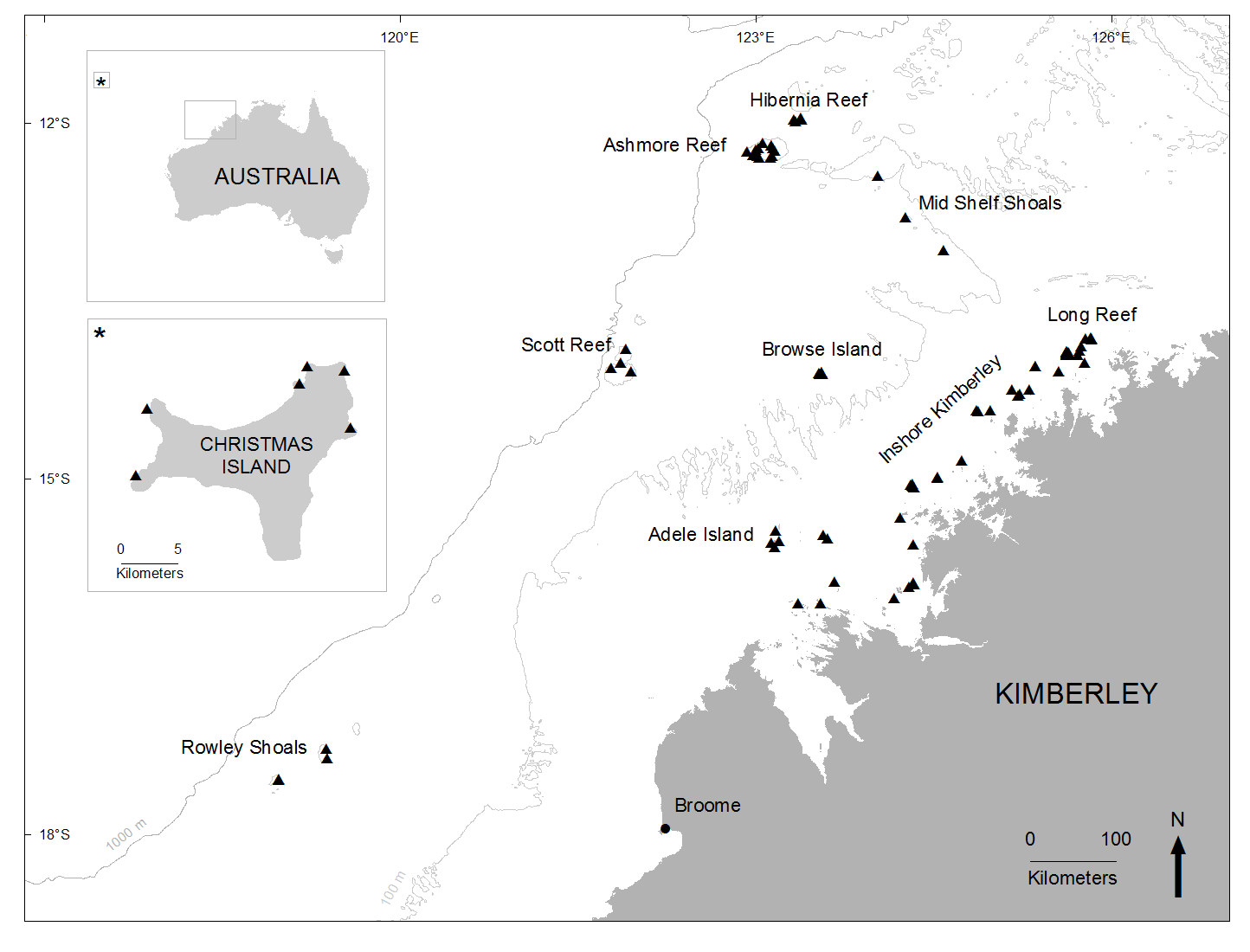
**Fig S1.** The regional distribution of *Heliopora* (triangles) in the Kimberley and Christmas Island which is an external Australian Territory in the eastern Indian Ocean approximately 1600 km from the Kimberley coast. Surveys of abundance were undertaken on replicate 15 m x 1 m belt transects (45-60 m^-1^ surveyed per site) undertaken at 0-20 m depth at 165 sites covering a total of 8.265 km^-1^.

**Fig S2.** The 20 most abundant species of reef-building coral recorded on belt transects in the intertidal (A) and subtidal (B) zones in the Kimberley. Shown is the number of individuals recorded on belt transects confirming that *Heliopora coerulea* is an important reef building species in both intertidal and subtidal habitats.

**
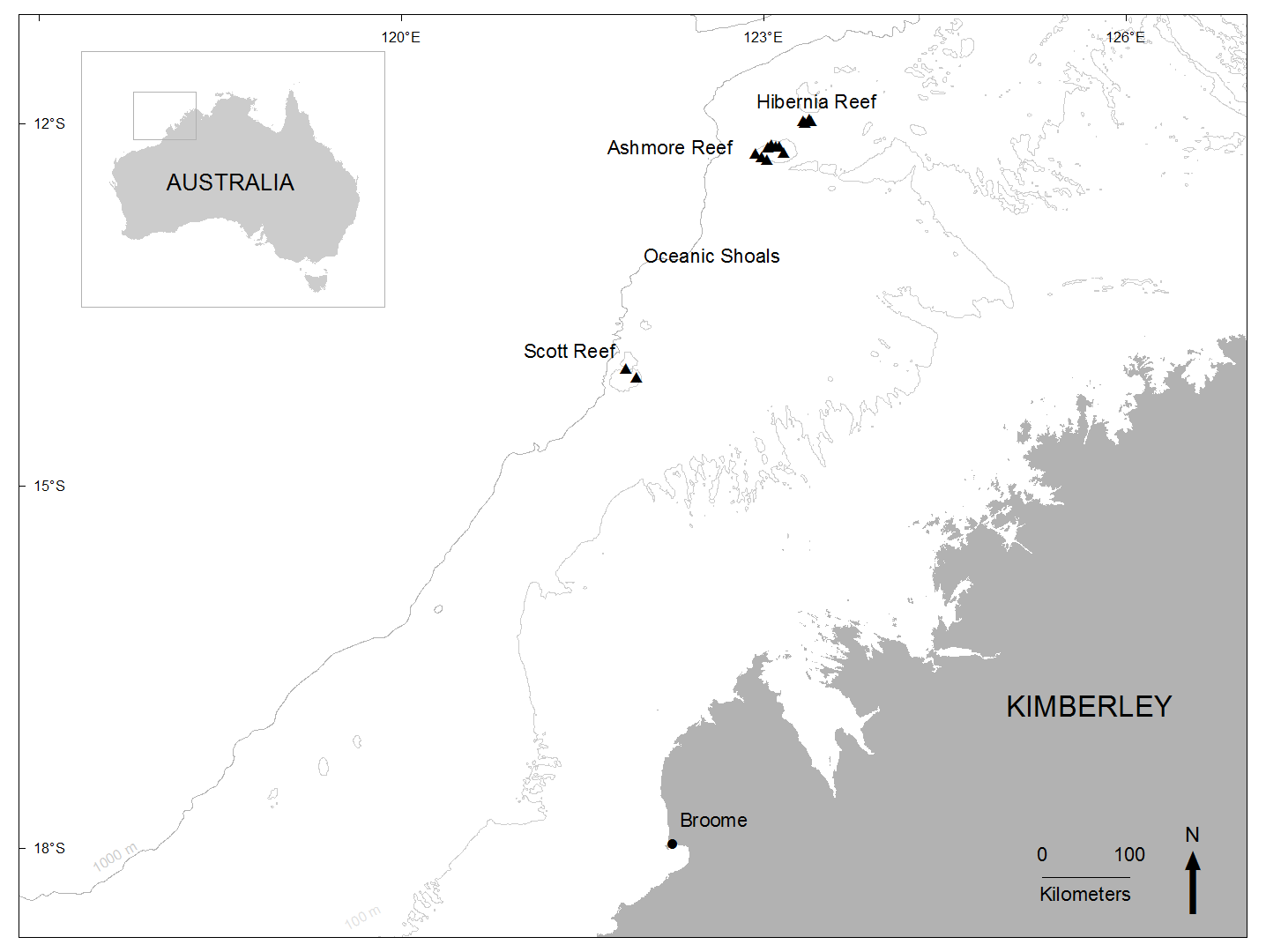
**

**Fig S3.** Distribution of *Heliopora hiberniana* sp. nov. Richards 2018 in the Kimberley, NW Australia (white and intermediate morphs).

**Fig S4.** Phylogenetic analysis using the nuclear *ITS2* gene confirms the divergence of *Heliopora hiberniana* sp. nov. from *Heliopora coerulea*.

**Fig S5.** Heatmap of *Symbiodinium* OTUs in *Heliopora* morphotypes. All OTUs derived using a 97% similarity threshold belonged to clade C *Symbiodinium*. The dominant OTU, C_OTU1_, represented by ITS2 type C1, grouped 95.2% of all sequences. C_OTU2_, represented by a novel ITS2 sequence associated with two intermediate colour morph colonies and accounted for 3.0% of all sequences. The remaining 41 clade C OTUs were in low abundance (<0.05% of reads). The colour (scale bar) represents the proportion of each OTU in the colony (after standardisation and square root transformation). Also, there was no significant difference in the *Symbiodinium* OTUs associated with different colour morphs of *Heliopora*.

**Table S1. Morphological characters and level of replication used for morphological analysis.**

| **Character** | **Replication** |
| --- | --- |
| Number of autopores per 5 mm^2^ | **3** |
| Autopore diameter [cm] | **8** |
| Autopore spacing [cm] | **8** |
| Coenchymal echinulations per 1 mm^2^ | **6** |
| Number of siphonopores [solenial tubes] per 1 mm^2^ | **6** |
| Number of elaborations per echinulation | **15** |
| Number of pseudosepta per autopore | **15** |
| Number of worm tubes per 5 mm^2^ | **3** |
| Diameter of tube worm openings [cm] | **7** |

**Table S2. Samples used for morphological analysis, reproductive and ITS2 phylogenetic analyses**

| **Sample Number** | **WAM accession number** | **Colour Morph** | **Gross Morphology** | **Collection Site** | **Co-ordinates** | **Depth** | **Morphological Analysis** | **ITS2 Phylogenetic Analysis** | **Reproductive Analysis** |
| --- | --- | --- | --- | --- | --- | --- | --- | --- | --- |
|  |  |  |  |  |  |  |  |  |  |
| **#511** | **89296** | blue | Columnar | West Montalivet Island Site 117 | 14°17.2998`S | 12m | Yes | No | No |
|  |  |  |  |  | 125°13.5336`E |  |  |  |  |
| **#32** | **89300** | blue | Columnar | NW Hibernia Reef Site 144 | 12°10.3782`S | 12m | Yes | Yes | Yes |
|  |  |  |  |  | 123°03.6696`E |  |  |  |  |
| **#187** | **89313** | blue | Columnar | North Scott Reef Site NCS | 13°54.363'S | 10m | Yes | No | No |
|  |  |  |  |  | 121°54.292'E |  |  |  |  |
| **#112** | **89304** | blue | Columnar | S Ashmore Reef Site 128 | 12°17.6100`S | 12m | Yes | No | No |
|  |  |  |  |  | 123°7.4238`E |  |  |  |  |
| **#135** | **66330** | blue | Columnar | E Ashmore Reef Site 135 | 12°14.6214`S | 12m | No | Yes | Yes |
|  |  |  |  |  | 122°14.6214`E |  |  |  |  |
| **#136** | **89307** | blue | Columnar | N Ashmore Reef Site 136 | 12°11.6688`S | 12m | No | Yes | Yes |
|  |  |  |  |  | 123°3.0090`E |  |  |  |  |
| **#119** | **89305** | blue | Columnar | Nth Ashmore Reef Site 126 | 12°11.0352`S | 12m | No | Yes | Yes |
|  |  |  |  |  | 123°6.0378`E |  |  |  |  |
| **#83** | **89302** | blue | Columnar | E Ashmore Reef Site 127 | 12°14.2368`S | 12m | No | Yes | Yes |
|  |  |  |  |  | 123°9.6024`E |  |  |  |  |
| **#143** | **89298** | blue | Columnar | SW Hibernia Reef Site 142 | 11°59.2890`S | 10m | No | Yes | Yes |
|  |  |  |  |  | 123°20.1510`E |  |  |  |  |
| **#34** | **66417 [Holotype]** | white | Branching | NW Hibernia Reef Site 144 | 11°58.4424`S | 12m | Yes | Yes | Yes |
|  |  |  |  |  | 123°19.3248`E |  |  |  |  |
| **#41** | **66411 [Paratype]** | white | Branching | NE Hibernia Reef Site 143 | 11°57.7002`S | 12m | Yes | Yes | Yes |
|  |  |  |  |  | 123°22.7148`E |  |  |  |  |
| **#36** | **66400 [Paratype]** | white | Branching | SW Hibernia Site 142 | 11°59.2890`S | 10m | Yes | Yes | Yes |
|  |  |  |  |  | 123°20.1510`E |  |  |  |  |
| **#43** | **66419 [Paratype]** | white | Branching | SW Hibernia Reef Site 142 | 11°59.2890`S | 10m | No | Yes | Yes |
|  |  |  |  |  | 123°20.1510`E |  |  |  |  |
| **#172** | **66329** | white | Branching | S Ashmore Reef Site 134 | 12°16.4868`S | 12m | Yes | No | Yes |
|  |  |  |  |  | 122°58.8768`E |  |  |  |  |
| **#129** | **66283** | white | Branching | Nth Ashmore Reef Site 126 | 12°11.0352`S | 12m | No | Yes | Yes |
|  |  |  |  |  | 123°6.0378`E |  |  |  |  |
| **#0** | **89297** | intermediate | Branching | NE Hibernia Reef Site 143 | 11°57.7002`S | 12m | Yes | No | No |
|  |  |  |  |  | 123°22.7148`E |  |  |  |  |
| **#122** | **89306** | intermediate | Branching | Nth Ashmore Reef Site 126 | 12°11.0352`S | 12m | Yes | No | No |
|  |  |  |  |  | 123°6.0378`E |  |  |  |  |
| **#73** | **89301** | intermediate | Branching | Nth Ashmore Reef Site 130 | 12°11.3088`S | 12m | Yes | No | No |
|  |  |  |  |  | 123°7.7322`E |  |  |  |  |
| **#174** | **89319** | intermediate | Branching | South Scott Reef SS1 | 14°1.424'S | 3m | No | Yes | No |
|  |  |  |  |  | 121°51.623'E |  |  |  |  |
| **#26** | **89299** | intermediate | Branching | SW Hibernia Reef Site 142 | 11°59.2890`S | 10m | No | Yes | No |
|  |  |  |  |  | 123°20.1510`E |  |  |  |  |
| **#40** | **66410** | intermediate | Branching | NE Hibernia Reef Site 143 | 11°57.7002`S | 12m | No | Yes | No |
|  |  |  |  |  | 123°22.7148`E |  |  |  |  |
| **#59** | **66397** | intermediate | Branching | Ashmore Reef Site 141 | 12°12.6126`S | 1m | No | Yes | No |
|  |  |  |  |  | 123°08.6436`E |  |  |  |  |
| **#39** | **66412** | intermediate | Branching | NW Hibernia Reef Site 144 | 12°10.3782`S | 12m | No | Yes | No |
|  |  |  |  |  | 123°03.6696`E |  |  |  |  |

**Table S3. Character and character state coding used for cladistics analysis.**

| **Character Number** | **Character** | **States** | **Coding** |
| --- | --- | --- | --- |
| 1 | Colour | Blue | 0 |
|  |  | White-blue streaks | 1 |
|  |  | White | 2 |
| 2 | Gross Morphology | columnar-upright fronds- nodular or encrusting with branches >15 mm diameter | 0 |
|  |  | thin branches <15 mm diameter | 1 |
| 3 | Max. number of elaborations per echinulation | few [up to 3] | 0 |
|  |  | many [more than 3] | 1 |
| 4 | Max. number of pseudosepta per autopore | moderate [12 or less] | 0 |
|  |  | many [13 or more] | 1 |
| 5 | Max. number of siphonopores (solenial tubes) per 1mm^2^ | moderate [up to 23] | 0 |
|  |  | many [24 or more] | 1 |
| 6 | Number of worm tubes per 5 mm^2^ | many [11 or more] | 0 |
|  |  | moderate [up to 10] | 1 |
|  |  | no worm tubes | 2 |
| 7 | Max. number of autopores per 5mm^2^ | moderate [15 or less] | 0 |
|  |  | many [over 15] | 1 |
| 8 | Min. autopore diameter | large [greater than 0.5 mm] | 0 |
|  |  | small [0.5 mm or less] | 1 |
| 9 | Max. autopore spacing | large [greater than 1.5 cm] | 0 |
|  |  | moderate [less than 1.5 cm] | 1 |
| 10 | Max. number of coenchymal echinulations per 1 mm^2^ | moderate [up to 23 ] | 0 |
|  |  | many [24 or more] | 1 |

**Table S4. Matrix of character codes used in cladistics analysis**

| **Characters** | **#511** | **#32** | **#187** | **#112** | **#41** | **#34** | **#36** | **#172** | **#0** | **#122** | **#73** |
| --- | --- | --- | --- | --- | --- | --- | --- | --- | --- | --- | --- |
| **1** | 0 | 0 | 0 | 0 | 2 | 2 | 2 | 2 | 1 | 1 | 1 |
| **2** | 0 | 0 | 0 | 0 | 1 | 1 | 1 | 1 | 1 | 1 | 1 |
| **3** | 0 | 0 | 0 | 0 | 1 | 1 | 1 | 1 | 1 | 1 | 1 |
| **4** | 0 | 1 | 0 | 0 | 1 | 1 | 1 | 1 | 1 | 1 | 1 |
| **5** | 0 | 0 | 0 | 1 | 1 | 1 | 1 | 1 | 1 | 1 | 1 |
| **6** | 1 | 2 | 0 | 0 | 2 | 2 | 2 | 2 | 2 | 1 | 0 |
| **7** | 0 | 0 | 0 | 0 | 1 | 1 | 0 | 1 | 1 | 1 | 0 |
| **8** | 0 | 0 | 0 | 0 | 1 | 0 | 1 | 1 | 1 | 1 | 1 |
| **9** | 0 | 0 | 0 | 0 | 1 | 0 | 0 | 1 | 1 | 1 | 0 |
| **10** | 0 | 0 | 0 | 0 | 1 | 1 | 1 | 1 | 1 | 1 | 1 |

**Table S5**. One-way analysis of variance (ANOVA) with Tukey’s HDS post-hoc comparisons of morphological measurements of ‘Blue’, ‘White’ and ‘Intermediate’ *Heliopora* groups.

| One-way ANOVA | | | n | | df | f-value | | | p-value | | |
| --- | --- | --- | --- | --- | --- | --- | --- | --- | --- | --- | --- |
| Number of autopores per 5 mm^2^ | | | 11 | | 2 | 2.973 | | | 0.108 | | |
| Autopore diameter | | | 11 | | 2 | 9.663 | | | **0.007** | | |
| Autopore spacing | | | 11 | | 2 | 2.609 | | | 0.134 | | |
| Coenchymal echinulations | | | 11 | | 2 | 6.544 | | | **0.021** | | |
| Number of siphonopores | | | 11 | | 2 | 9.587 | | | **0.008** | | |
| Elaborations per echinulation | | | 11 | | 2 | 61.863 | | | **<0.001** | | |
| Pseudosepta per autopore | | | 11 | | 2 | 6.537 | | | **0.021** | | |
|  | | |  | |  |  | | |  | | |
| Tukey’s HSD post-hoc comparisons  [p-values] | | | Blue | | | White | | | Intermediate | |  |
| Autopore diameter | Blue | | - | | | | - | | | - |  |
|  | White | | **0.01** | | | | - | | | - |  |
|  | Intermediate | | **0.021** | | | | 0.967 | | | - |  |
| Coenchymal echinulations | Blue | | - | | | | - | | | - |  |
|  | White | | 0.098 | | | | - | | | - |  |
|  | Intermediate | | **0.019** | | | | 0.439 | | | - |  |
| Siphonopores | Blue | | - | | | | - | | | - |  |
|  | White | | **0.019** | | | | - | | | - |  |
|  | Intermediate | | **0.011** | | | | 0.791 | | | - |  |
| Elaborations per echinulation | Blue | | - | | | | - | | | - |  |
|  | White | | **<0.001** | | | | - | | | - |  |
|  | Intermediate | | **<0.001** | | | | **0.047** | | | - |  |
| Pseudosepta per autopore | Blue | | - | | | | - | | | - |  |
|  | White | | **0.03** | | | | - | | | - |  |
|  | Intermediate | | **0.042** | | | | 1 | | | - |  |

**Table S6.** Independent samples T-test comparing the means of morphological measurements taken from SEM images between ‘Blue’ and ‘White’ corals when ‘White’ and ‘Intermediate’ corals were pooled together. Where equality of variance assumption was not met *i.e*. ‘autopore diameter’, Welch’s T-test was used.

| Independent Samples T-test | n | df | t-value | p-value |
| --- | --- | --- | --- | --- |
| Number of autopores per 5 mm^2^ | 11 | 9 | -2.364 | **0.042** |
| Autopore spacing (cm) | 11 | 9 | 2.411 | **0.039** |
| Number of coenchymal echinulations per 1cm^2^ | 11 | 9 | -3.263 | **0.010** |
| Number of siphonopores per 1 cm^2^ | 11 | 9 | -4.470 | **0.002** |
| Number of elaborations/echinulation | 11 | 9 | -7.943 | **<0.001** |
| Pseudosepta per autopore | 11 | 9 | -3.835 | **0.004** |
|  |  |  |  |  |
| Welch T-test |  |  |  |  |
| Autopore diameter | 11 | 3.631 | -3.728 | **0.024** |
|  |  |  |  |  |

**Table S7** – Markers, Primers and PCR conditions used in this study

| Gene | Marker | Marker Sequence | PCR Conditions |
| --- | --- | --- | --- |
| *COI* | COII-8068F | CCATAACAGGACTAGCAGCATC | 95°C for 3mins, followed by 35 cycles of 95°C for 40 secs, 58°C for 90 sec, 68°C for 50 sec followed by an extension time of 68°C for 5 mins. |
|  | COI-OCTr | ATCATAGCATAGACCATACC |  |
| *msh1* | ND42599F | GCCATTATGGTTAACTATTAC | 94°C for 3mins, followed by 35 cycles of 94°C for 30 secs, 52°C for 30 sec, 72°C for 30 sec followed by an extension time of 72°C for 5 mins. |
|  | mut3458R | TSGAGCAAAAGCCACTCC |  |
| *ITS2* - host | 5.8S-436 | AGCATGTCTGTCTGAGTGTTGG | 94°C for 2mins, followed by 30 cycles of 94°C for 30 secs, 56°C for 45 sec, 72°C for 45 sec followed by an extension time of 72°C for 5 mins. |
|  | 28S-663 | GGGTAATCTTGCCTGATCTGAG |  |
| *ITS2* - symbiont | ITSD | GTGAATTGCAGAACTCCGTG | 95°C for 5mins, followed by 35 cycles of 95°C for 30 secs, 52°C for 30 sec, and 72°C for 45 sec, followed by a final extension time of 10 mins at 72°C. |
|  | ITS2rev2 | CCTCCGCTTACTTATATGCTT |  |

**Table S8.** Site coordinates of population genetic samples

| **Abbreviation** | **Location** | **n** | **Colour** | **Station Details** | **Co-ordinates** | | **Depth** |
| --- | --- | --- | --- | --- | --- | --- | --- |
| Hib | Hibernia Reef, Offshore Kimberley | 30 | White | Stn 144 | 11°58.4424`S | 123°19.3248`E | 12m |
| HBL | Hibernia Reef, Offshore Kimberley | 27 | Blue | Stn 142 | 11°59.2890`S | 123°20.1510`E | 12m |
| ASH | Ashmore Reef, Kimberley offshore Atoll | 19 | Blue | Stn 138 | 12°16.7148`S | 123°01.0266`E | 6m |
| BRS | Browse Island, Kimberley Midshelf Island | 30 | Blue | Stn 104 | 14°06.4848`S | 123°33.3084`E | 0m |
| ECH | Echuca Reef, Kimberley Midshelf Shoal | 19 | Blue | Stn 107 | 13°53.7810`S | 123°53.6856`E | 20m |
| LNG | Long Reef, northern Kimberley nearshore reef | 24 | Blue | Stn 43 | 13°55.2930`S | 125°43.9608`E | 12m |
| CHM | Champagny Island, Southern Kimberley nearshore reef | 29 | Blue | Stn 63 | 15°19.8444`S | 124°13.0152`E | 0m |
| XMAS | Rocky Point, Christmas Island | 12 | Blue | Rocky Point | 10°30.068`S | 105°32.317`E | 12m |

**Table S9.** Heterozygosity, F statistics and Polymorphism in Western Australian populations of *Heliopora* based on microsatellite data. Sample Size (N), No. Alleles (Na), No. Effective Alleles (Ne), Information Index (I), Observed Heterozygosity (Ho), Expected (He) and Unbiased Expected Heterozygosity (UHe), and Fixation Index (F).
